# Total laparoscopic total gastrectomy and distal esophagectomy combined with reconstruction by transhiatal esophagojejunal Roux-en-y mediastinal anastomosis for Siewert II AEG

**DOI:** 10.1186/s13019-023-02453-5

**Published:** 2023-11-22

**Authors:** Qifan Yin, Guibin Zhang, Peng Qie, Shaohui Han, Lijun Liu

**Affiliations:** Thoracic Surgery, Hebei General Hospital, No 348,West He-Ping Road, Xinhua District, Shijiazhuang, 050000 Hebei Province China

**Keywords:** Adenocarcinoma of the esophagogastric junction (AEG), Total laparoscopic Surgery, Mediastinal anastomosis

## Abstract

**Purpose:**

The optimal procedure is still controversial about Siewert type II AEG, We are attempt to explore the efficacy and feasibility of total laparoscopic total gastrectomy and distal esophagectomy combined with reconstruction by transhiatal esophagojejunal Roux-en-y mediastinal anastomosis for Siewert type II AEG.

**Method:**

Data of patients with Siewert type II AEG who received total laparoscopic total gastrectomy and distal esophagectomy combined with reconstruction by transhiatal esophagojejunal Roux-en-y mediastinal anastomosis in the Hebei General Hospital were collected from October 2020 to October 2021, The operation time, surgical blood loss, the number of dissected lymph nodes, duration of drainage tube, the length of stay in ICU, the resume oral feeding time, the length of postoperative hospital stay, postoperative complications and other related indicators of the patients were collected to evaluate the safety and feasibility of this operation.

**Result:**

A total of 17 patients received total laparoscopic total gastrectomy and distal esophagectomy combined with reconstruction by transhiatal esophagojejunal Roux-en-y mediastinal anastomosisin the treatment of Siewert type II AEG were analyzed in our research. The mean operation time was 253 ± 24.8 min (196–347 min); The median surgical blood loss was 250 ml (20–2400 ml); The average number of dissected lymph nodes were 28 ± 4.6 (17–36); The median duration of drainage tube was 5 days (3–7days); The median length of stay in ICU was 18 h(10–34 h); The median time of resume oral feeding was 6 days (5–7days); The median postoperative hospital stay was 11 days (8–15 days). Among the all enrolled patients, one patient underwent the conversion to laparotomy due to the massive intraoperative bleeding, one patient developed anastomotic stenosis at jejunum side-to-side anastomosis on the first month after surgery, there was no case of death during the operation and postoperative anastomotic fistula. All patients achieved R0 resection with an average distance of 6 cm (4–8.5 cm) from the upper margin of the tumor to the resection margin.

**Conclusion:**

The operation of total laparoscopic total gastric and distal esophagectomy combined with reconstruction by transhiatal esophagojejunal Roux-en-y mediastinal anastomosis is technically feasible and sufficiently safe in the treatment of Seiwert type II AEG from our primary clinical experience. This procedure could be one of the alternatives for the radical treatment of Siewert type II AEG.

## Introduction

In the last two decades, the incidence of adenocarcinoma of the esophagogastric junction (AEG) has been increasing rapidly worldwide [1]. AEG tumors were commonly classified into three types, from type I to type III, according to the position of the center of the main tumor referring to the esophagogastric junction (EGJ) by Siewert et al. research in 1998 [2]. Up to now, Complete tumor resection (R0) with adequate lymph node dissection has been regarded as the only possible radical treatment for all types of AEG with a curative intent. Due to its specific anatomical location, there are many surgical approaches for AEG, including thoracoabdominal esophagectomy, left transthoracic esophagectomy, transhiatal esophagectomy, combined thoracoabdominal esophagectomy [3–5]. No major controversies exist about the surgical strategies for AEG type I and III tumors. The majority of scholars currently advocate that right transthoracic esophagectomy combined with mediastinal lymph node dissection should be recommended to Siewert type I AEG [6–8]. The biological behavior and lymph node metastasis of Siewert type III AEG are more similar to gastric cancer, which lead to many similarities with gastric cancer in treatment. Currently, most specialists claim that Siewert type III AEG tumors should be performed transabdominal total gastrectomy and distal esophagectomy alongside with D2 lymphadenectomy [9, 10]. However, there is still controversy regarding the optimal surgical approach for Siewert type II AEG, with some surgeons preferring a abdomino-transhiatal approach while others favor a transthoracic approach [11, 12]. Some studies have shown that a thoracoabdominal approach is needed to achieve sufficient mediastinal and abdominal lymphadenectomy as well as negative resection margins [13, 14]. On the other hand, there are indications for higher morbidity rates after thoracoabdominal surgery and poor outcome of patients even complete resection and radical lymphadenectomy [4, 15].

Recently, The operation of total laparoscopic total gastrectomy and distal esophagectomy combined with reconstruction by transhiatal esophagojejunal Roux-en-y mediastinal anastomosis has been accomplished for several Siewert II AEG patients in our center and achieve promising short-term efficacy. In this study, we report the technical details and clinical experience of this surgical approach for Siewert type II AEG.

## Materials and methods

### Patients

17 patients with Siewert type II AEG were collected into our study from October 2020 to October 2021, all of whom had underwent total laparoscopic total gastrectomy and distal esophagectomy combined with reconstruction by transhiatal esophagojejunal Roux-en-y mediastinal anastomosis in our department in Hebei General Hospital. 11 male and 6 female, age from 50 to 74, average age 62 ± 3.7. Preoperative diagnosing and staging were based on gastroscopy, endoscopic ultrasonography, esophagogram, chest and abdomen enhanced computed tomograpy, ultrasound of cervical lymph nodes and so on. All patients had definite preoperative pathological findings and were diagnosed as Siewert type II AEG. The staging in all patients was ranging stage I to stage III, beyond the indication range for endoscopic mucosal resection or endoscopic submucosal dissection. The length of lesion was estimated to be ≤ 4 cm in all cases, The length of tumor invasion to the lower esophagus did not exceed 2 cm. Surgery was approved after the informed consent was obtained from the patients or authorized family members. The basic characteristic of enrolled 17 patients was shown in Table 1.

**Table 1:** The basic characteristics of the patients

Patient’s characteristic	Number (n = 17)
Gender	
Male	11
Female	6
Age	
Average	62 ± 3.7
Range	50–74
Smoking status	
Smoking	7
Non-smoking	10
BMI	24.12 ± 2.70 kg/m^2^
Length of lesion	
Average	3.5 cm
Range	2-4 cm
Clinical staging	
cT2N0M0	3
cT3N0M0	5
cT2N1M0	4
cT3N1M0	5

### Surgical procedure

Single lumen endotracheal tube was intubated after successfully general anesthesia. The patient was placed in the supine position with legs spread and dorsal elevated. The surgeon stands on the patient’s right side, the assistant occupies the patient’s left side, and the lens holder stands between the patient’s legs. The incisions of five ports for the laparoscopic operation were made as follows: one 1-cm incision was made above the umbilicus and used as the laparoscopic port, two1-cm incisions were made at outer edge of right rectus abdominis at the level of the umbilicus and below the right costal margin of the midclavicular line and used as the main operative ports. Another two 5-mm incisions were made at outer edge of left rectus abdominis and under xiphoid and used as the assisting ports.

**Fig. 1:**
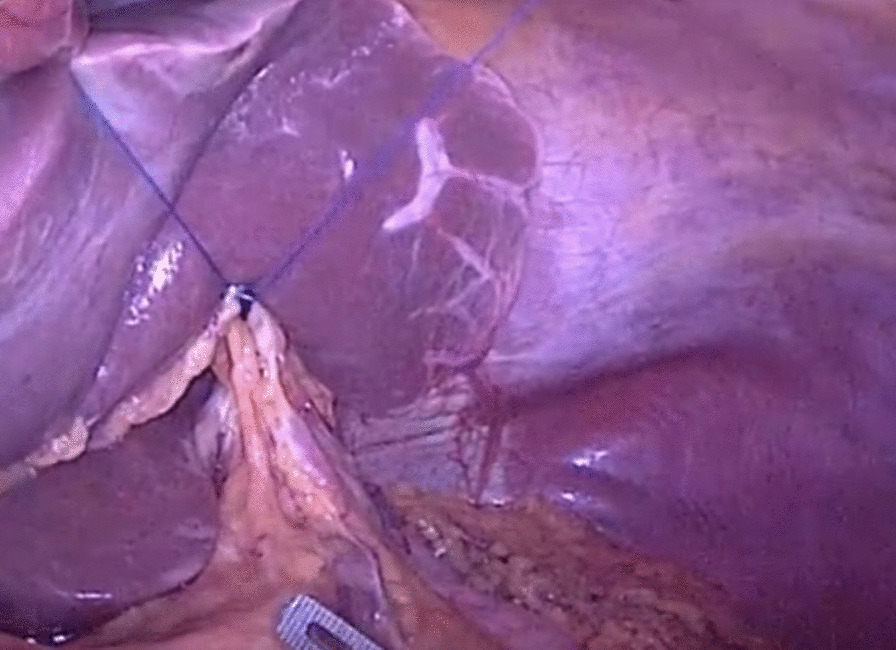
The suspension of the liver

Carbon dioxide was injected into the abdomen with a pressure of 12–14 mmHg to cause artificial pneumoperitoneum. The liver was retracted by traction forceps after complete peritoneal exploration. The lesser omentum was dissected using the conventional method. As shown in Fig. 1, the liver was retracted using purse string combined with hemo-lock suspension. The vascularless area near the transverse colon of the omentum majus was found. The greater omentum was dissected by ultrasonic knife along the upper edge of the transverse colon in the fascial dissection space using conventional method, and then mobilized right to the hepatic flexure of colon. The anterior layer of the transverse mesocolon is stripped off the pancreatic capsule, The root of right gastroomentum vessel was dissected on the superficial surface of the pancreatic head, divided by ultrasonic scalpel after hemo-lock ligation. Subpyloric lymph nodes and adipose tissue were removed. The lower margin of duodenal bulb was mobilized. The omentum majus was mobilized left to the splenic flexure of colon, the left gastroomentum vessel was identified and divided by ultrasonic scalpel, the gastrosplenic ligaments and all short gastric vessels were dissected using ultrasonic scalpel. The omentum is dissected away from the colon and the spleen. The phrenoesophageal membrane was subsequently divided to expose the abdominal esophagus and left diaphragmatic foot. The hepatoduodenal ligament was opened and the right gastric artery, proper hepatic artery and common hepatic artery were found. The right gastric artery was clipped and divided at the level of its root. The superior pyloric lymph nodes and intrahepatic duodenal ligament lymph nodes were removed. The gastric body was pulled up, the adhesion between the posterior wall of the stomach and pancreas was separated, the gastric pancreatic fold was lifted, the left gastric artery and gastric coronary vein were identified and dissociated, which were ligated with hemo-lock and divided using ultrasonic scalpel. The lymph nodes of station 7, 8, 9 and 11 were removed. The lymphatic and adipose tissue between the lesser omentum and right diaphragmatic foot was removed using ultrasonic scalpel in order to fully mobilize the lesser curvature of stomach. The antrum of the stomach was stirred up and the duodenum was cut with a linear stapler at 2–3 cm below the pylorus. The assistant clamped the stump of duodenum and pulled the stomach to the left, and the surgeon separated all adhesions so that the stomach and abdominal esophagus were fully mobilized.

**Fig. 2:**
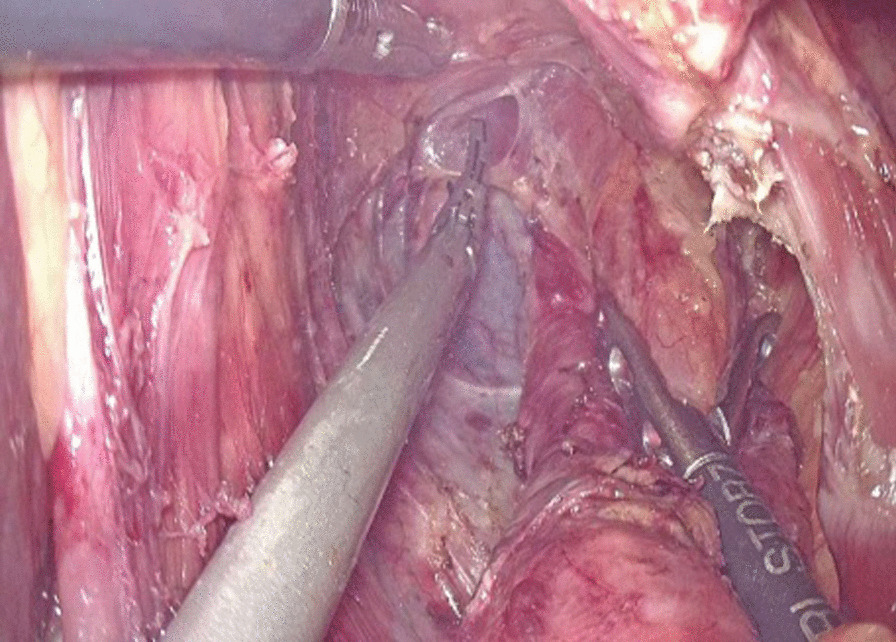
The mobilization of the lower esophagus

**Fig. 3:**
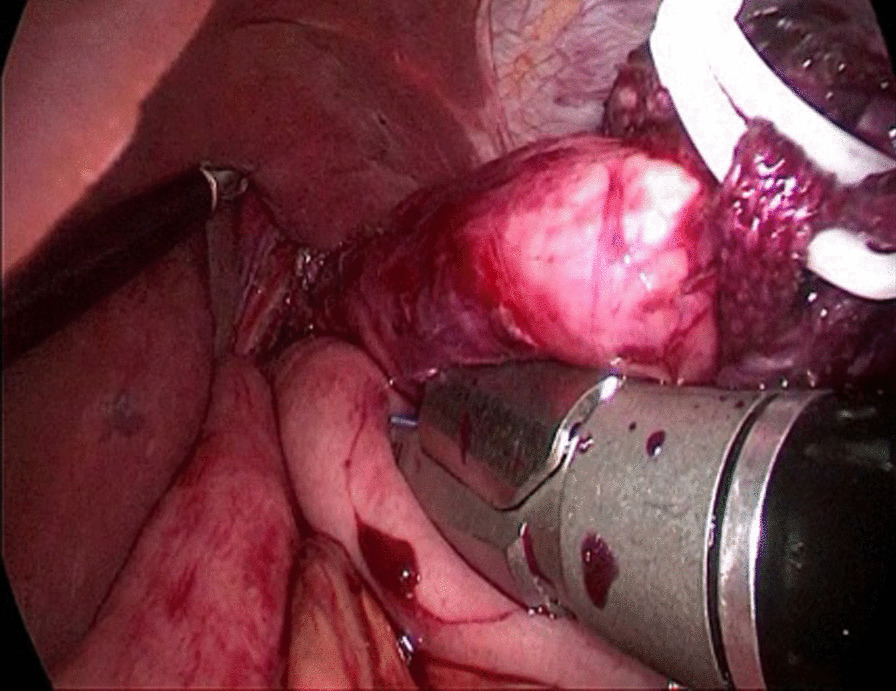
The side-to-side anastomosis of esophagus and jejunum

**Fig. 4:**
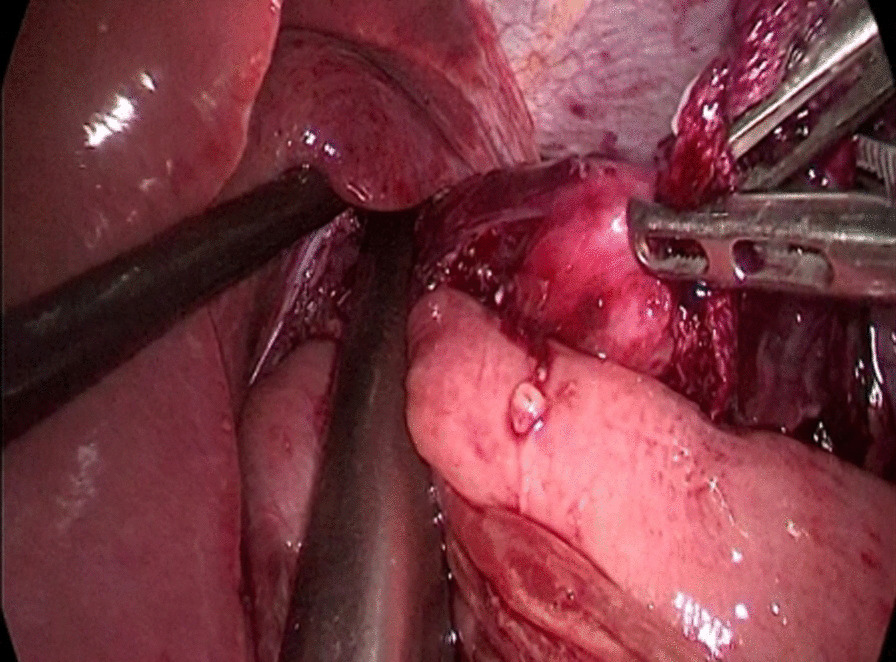
The closure of common esophagojejunal opening

**Fig. 5:**
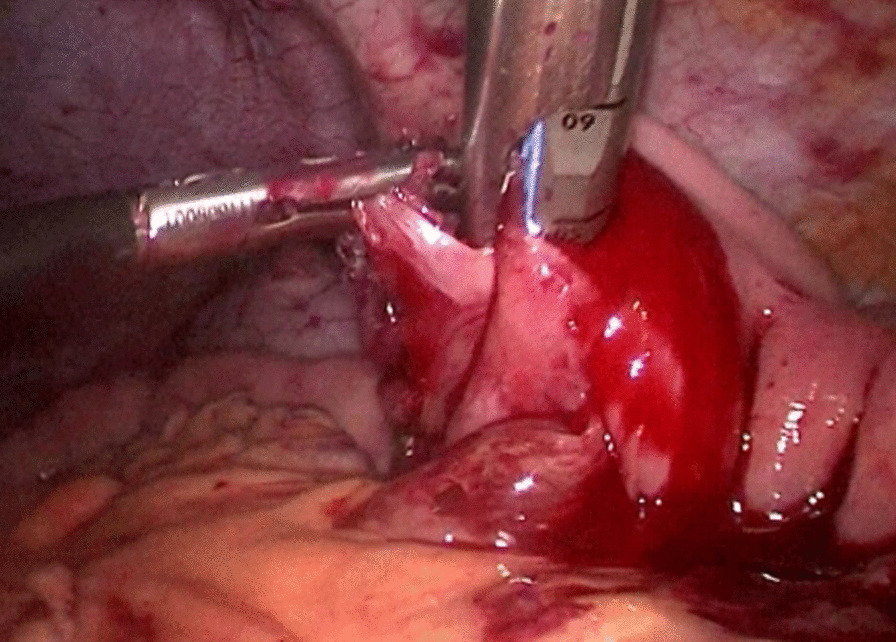
The jejunum side-to-side anastomosis

**Fig. 6:**
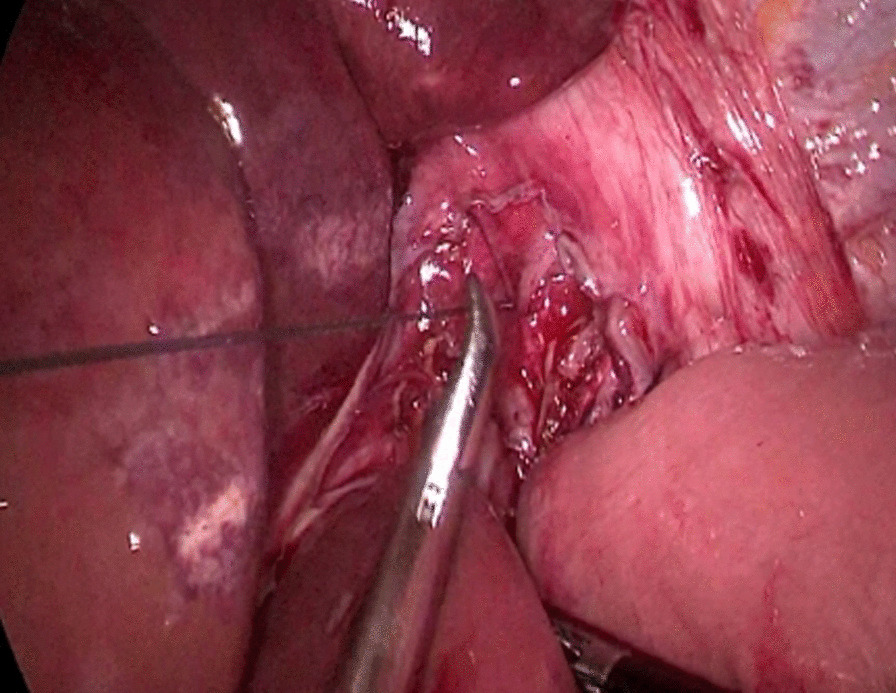
The closure of esophageal hiatus

The abdominal esophagus was encircled using cotton tape, which was pulled to stretch the esophageal wall by assistant. The esophageal hiatus was opened with an ultrasonic scalpel. The lower esophagus in the mediastinum was then mobilized upward from the hiatus until we reached the level of the carina and fully mobilized to obtain a sufficient proximal margin from the tumor (Fig. 2). The pericardial lymph nodes and extended lower mediastinal lymph nodes (including lymph nodes around the subcarinal and inferior pulmonary) were dissected. The assistant pulled the esophageal band to prevent esophageal retraction and acquire sufficient margin, A small incision was made on the right side of the lower esophageal wall with ultrasonic scalpel. A another small incision was made on the side wall of jejunum at the about 15 cm from the distal end of the Treitz ligament. Part of the mesangium here was dissected to lift it up, the two arms of the 60 mm linear stapler were inserted into jejunum and esophagus from the holes of jejunum and esophagus, respectively, to make a side-to-side anastomosis, which was type “π” anastomosis (Fig. 3). The common esophagojejunal opening was closed with a 60 mm linear stapler and then reinforced with stapler line reinforcement (Fig. 4). At the same time, the esophagus and jejunum were transected and the specimen was freed. This completes the esophagojejunostomy. A small incision was made at the proximal jejunum and the jejunum about 40–60 cm from the distal end of the anastomosis, respectively. The twoarms of the 60 mm linear stapler were inserted into the intestinal lumen respectively to make jejunum side-to-side anastomosis (Fig. 5). The common jejunum incision was closed with the 60 mm linear stapler and the anastomosis was reinforced with sutures. Esophageal hiatus was closed (Fig. 6). A 5-cm subxiphoid vertical incision was made, through which the specimen was pulled out. Two drainage tubes were placed at the anastomosis after adequate hemostasis. The operation was completed.

## Results

The operation time, surgical blood loss, the number of dissected lymph nodes, duration of drainage tube, the length of stay in ICU, the resume oral feeding time, the length of postoperative hospital stay, postoperative complications were collected by the third author after the operation. A total of 17 patients have successfully completed the surgery of total laparoscopic total gastrectomy and distal esophagectomy combined with reconstruction by transhiatal esophagojejunal Roux-en-Y mediastinal anastomosis for Siewert type II AEG. There was one case of conversion to laparotomy due to massive intraoperative bleeding and no case of death during the operation, No anastomotic fistula occurred, One patient developed anastomotic stenosis at jejunum side-to-side anastomosis on the first month after surgery, resulting in the obstruction of the input loop. Undergoing the second operation, the patient died within a short period after surgery. The other patients were safely discharged without any serious complications.

Among the all enrolled 17 patients, the mean operation time was 253 ± 24.8 min (196–347 min); The median surgical blood loss was 250 ml (20–2400 ml); The average number of dissected lymph nodes were 28 ± 4.6 (17–36); The median duration of drainage tube was 5 days (3–7days); The median length of stay in ICU was 18 h (10–34 h); The median time of resume oral feeding was 6 days (5–7days); The median length of postoperative hospital stay was 11 days (8–15 days); The details of postoperative indicators and complications are shown in Tables 2 and 3. The R0 resection rate was 100% from the postoperative pathological report. The average distance between the upper margin of the tumor and the surgical margin was 6 cm (4–8.5 cm).

**Table 2:** Analysis of postoperative indicators

Postoperative observed indicator	Data
The operation time(min)	
Average	253 ± 24.8
Range	196–347
Surgical blood loss(ml)	
Median	250
Range	20–2400
Number of dissected lymph nodes	
Average	28 ± 4.6
Range	17–36
Duration of drainage tube(day)	
Median	5
Range	3–7
Length of ICU stay (h)	
Median	18
Range	10–34
Time of resume oral feeding (day)	
Median	6
Range	5–7
Postoperative hospital stay (day)	
Median	11
Range	8–15

**Table 3:** Postoperative complications

Complications	Number (%)
Anastomotic fistula	0 (0)
Anastomotic stenosis	1 (6)
Intraoperative massive bleeding	1 (6)
Postoperative massive bleeding	0 (0)
Postoperative pneumonia	0 (0)
Re-operation	1 (6)
Perioperative death	0 (0)

## Discussion

After decades of research progress, the operation approaches of AEG include transabdominal approach, transthoracic approach, combined thoracoabdominal approach. However, different surgical methods have different pros and cons. Clinical studies have shown that the transabdominal approach is superior to the transthoracic approach for Siewert type II AEG patients [9, 16, 17].The study of The Japanese Clinical Oncology Group (JCOG9502) proved that the transhiatal approach was superior to the left thoracotomy approach in the treatment of Seiwert type II and III AEG tumors invading lower esophagus less than 3 cm, which could reduce postoperative complications [3]. In this study, we consider that the surgery of total laparoscopic total gastrectomy and distal esophagectomy combined with reconstruction by transhiatal esophagojejunal Roux-en-y mediastinal anastomosis can be one of the alternatives of radical operation for Siewert type II AEG, especially for AEG with lesion length ≤ 4 cm. Based on our previous surgical experience of inflatable mediastinoscopy combined with laparoscopy for radical esophagectomy in esophageal squamous cell carcinoma. A total of 17 patients have been performed this novel operation of total laparoscopic total gastrectomy and distal esophagectomy combined with reconstruction by transhiatal esophagojejunal Roux-en-y mediastinal anastomosis in the treatment of their Seiwert type II AEG in our center.

According to a number of current researches and Japanese gastric cancer treatment guidelines [1, 10, 18–20], the biological behavior and pathological features of Siewert type II AEG in Asian patients bear more resemblance to gastric cancer than that of esophageal cancer. The purpose of surgical treatment for Siewert II AEG is to obtain sufficient negative margin and radical mediastinal and abdominal lymph nodes dissection in consideration of current study [20]. Previous literatures have shown that the combined thoracoabdominal approach is the most common surgical approach for Siewert type II AEG, and have fully compared the pros and cons between them [10, 13, 18, 19]. The operation method of Ivor-Lewis selects the advantages of transthoracic or transabdominal approach operation for Siewert type II AEG, enabling to fully mobilize esophagus and stomach, ensuring no residual cancer cells in surgical margin, completely dissecting abdominal and mediastinal lymph nodes, achieving radical resection. However, The research results showed that the combined thoraco-abdominal approach not only failed to improve the long-term survival of the patients, but also increased the perioperative complications and mortality [4, 15].The main reason is because the Ivor-Lewis surgery has high requirements on cardiopulmonary function, longer operative time, larger surgical trauma, more postoperative complications.

The anastomotic method of this operation was linear anastomosis in our study. The main characteristic of mediastinal π-shaped esophagojejunostomy was three-in-one effect. Three procedures—esophageal division, common entry hole closure, and jejunal division—were performed during a single stapling after esophagojejunum side to side anastomosis [21]. In addition, The esophagogastric junction was constricted by a esophageal band to prevent gastric retraction and facilitate downward traction of the esophagus, which could acquire sufficient surgical margin, higher anastomotic position, achieve mediastinal esophagojejunostomy, avoid residual cancer cells in margin. Intraoperatively, the common entry hole of π-shaped esophagojejunostomy can be used to check whether there is tumor invasion or residue at the esophageal resection margin, further reducing the possibility of positive esophageal resection margin. The liver was retracted and exposed using purse string combined with hemo-lock suspension, which could free assistant’s hand. The pylorus proximal end was tracted leftward after initial division of pylorus using linear stapler by assistant, which made it easier to mobilize the stomach and avoided unnecessary injury. The operation of total laparoscopic total gastrectomy and distal esophagectomy combined with reconstruction by transhiatal esophagojejunal Roux-en-y mediastinal anastomosis avoided the complications caused by transthoracic surgical approach, had low requirements for cardiopulmonary function, especially for patients with total thoracic atresia and elderly patients. And total laparoscopic operation has lower cardiopulmonary function impairment, less postoperative complication, shorter abdominal incision, lighter postoperative pain, which can not only reduce the use of analgesics, but also help patients to get out of bed earlier, accelerate the postoperative recovery of patients. Compared to traditional laparoscopic surgery, our novel operation can obtain adequate upper surgical margin and sufficient mediastinal lymph nodes, and reduce the risk of postoperative recurrence. In addition, total gastrectomy can reduce the occurrence of postoperative acid reflux, heartburn and other uncomfortable symptoms.

The anastomotic fistula may be considered to be the most important and potentially life-threatening complication for this operation, There was no case occurring anastomotic fistula in our study. One patient developed anastomotic stenosis at jejunum side-to-side anastomosis on the first month after surgery, resulting in the obstruction of the input loop. The reason may be related to the closure of the jejunum common entry hole using linear stapler, which might be avoided to use barbed wire to close common incision. In addition, one patient suffered massive intraoperative bleeding and was transferred to laparotomy. The reason was severe external invasion of the tumor, which was closely related to the spleen. Intraoperative splenic artery laceration caused massive hemorrhage. The patient completed the operation successfully after laparotomy, while the other patients did not suffer serious complications and were discharged successfully.

Some limitations have to be addressed in our study. Firstly, this operation has relatively high requirements for the surgeon, demanding the surgeon to have a proficiency general surgery foundation. Secondly, this study was a single-arm study and did not conduct randomized control with traditional surgery. In addition, since the study was not conducted for a long time, The long-term follow-up of all enrolled patients was not acquired, so the survival data of patients are temporarily unavailable. We need to include more patients in the study and conduct long-term follow-up to obtain more substantial and reliable data to demonstrate the feasibility and safety of total laparoscopic total gastric and distal esophagectomy combined with reconstruction by transhiatal esophagojejunal Roux-en-y mediastinal anastomosisfor Siewert type II AEG in the future.

## Conclusion

The operation of total laparoscopic total gastric and distal esophagectomy combined with reconstruction by transhiatal esophagojejunal Roux-en-y mediastinal anastomosis is technically feasible and sufficiently safe in the treatment of Seiwert type II AEG from our primary clinical experience. This procedure could be one of the alternatives for the radical treatment of Siewert type II AEG. The large-scale prospective clinical trials may be needed to further assess the the efficacy and feasibility of this surgical method in the future.

## Data Availability

The datasets used and analyzed during the current study are available from the corresponding author on reasonable request.

## References

[1] Hasegawa S, Yoshikawa T (2010). Adenocarcinoma of the esophagogastric junction: incidence, characteristics, and treatment strategies. Gastric Cancer.

[2] Siewert JR, Stein HJ (1998). Classification of adenocarcinoma of the oesophagogastric junction. Br J Surg.

[3] Sasako M, Sano T, Yamamoto S, Sairenji M, Arai K, Kinoshita T, Nashimoto A, Hiratsuka M (2006). Japan Clinical Oncology G: Left thoracoabdominal approach versus abdominal-transhiatal approach for gastric cancer of the cardia or subcardia: a randomised controlled trial. Lancet Oncol.

[4] Ott K, Bader FG, Lordick F, Feith M, Bartels H, Siewert JR (2009). Surgical factors influence the outcome after Ivor-Lewis esophagectomy with intrathoracic anastomosis for adenocarcinoma of the esophagogastric junction: a consecutive series of 240 patients at an exper ienced center. Ann Surg Oncol.

[5] Orringer MB, Marshall B, Iannettoni MD (1999). Transhiatal esophagectomy: clinical experience and refinements. Ann Surg.

[6] Hulscher JB, van Sandick JW, de Boer AG, Wijnhoven BP, Tijssen JG, Fockens P, Stalmeier PF, ten Kate FJ, van Dekken H, Obertop H (2002). Extended transthoracic resection compared with limited transhiatal resection for adenocarcinoma of th e esophagus. N Engl J Med.

[7] Omloo JM, Lagarde SM, Hulscher JB, Reitsma JB, Fockens P, van Dekken H, Ten Kate FJ, Obertop H, Tilanus HW, van Lanschot JJ (2007). Extended transthoracic resection compared with limited transhiatal resection for adenocarcinoma of th e mid/distal esophagus: five-year survival of a randomized clinical trial. Ann Surg.

[8] von Rahden BH, Stein HJ, Siewert JR (2006). Surgical management of esophagogastric junction tumors. World J Gastroenterol.

[9] Sauvanet A, Mariette C, Thomas P, Lozac’h P, Segol P, Tiret E, Delpero JR, Collet D, Leborgne J, Prad¨¨re B (2005). Mortality and morbidity after resection for adenocarcinoma of the gastroesophageal junction: predicti ve factors. J Am Coll Surg.

[10] Japanese Gastric Cancer A (2017). Japanese gastric cancer treatment guidelines 2014 (ver. 4). Gastric Cancer.

[11] Lordick F, Mariette C, Haustermans K, Obermannov¨¢ R, Arnold D, Committee EG (2016). Oesophageal cancer: ESMO Clinical Practice guidelines for diagnosis, treatment and follow-up. Ann Oncol.

[12] Porschen R, Fischbach W, Gockel I, Hollerbach S, Hulscher A, Jansen PL, Miehlke S, Pech O, Stahl M, Thuss-Patience P (2019). S3-Leitlinie Diagnostik und Therapie der Plattenepithelkarzinome und Adenokarzinome des Ösophagus. Z Gastroenterol.

[13] Deng JY, Liang H (2014). Adenocarcinoma of esophagogastric junction. Chin J Cancer Res.

[14] Kodama I, Kofuji K, Yano S, Shinozaki K, Murakami N, Hori H, Takeda J, Shirouzu K (1998). Lymph node metastasis and lymphadenectomy for carcinoma in the gastric cardia: clinical experience. Int Surg.

[15] Yonemura Y, Tsugawa K, Fonseca L, Fushida S, Matsumoto H, Ninomiya I, Sugiyama K, Fujimura T, Nishimura G, Miwa K (1995). Lymph node Metastasis and surgical management of gastric cancer invading the esophagus. Hepatogastroenterology.

[16] Carboni F, Lorusso R, Santoro R, Lepiane P, Mancini P, Sperduti I, Santoro E (2009). Adenocarcinoma of the esophagogastric junction: the role of abdominal-transhiatal resection. Ann Surg Oncol.

[17] Gianotti L, Braga M, Landoni L, Mari G, Scaltrini F, Di Castelnuovo A, Di Carlo V (2003). Outcome of patients with cancer of the esophagogastric junction in relation to histology and surgical strategy. Hepatogastroenterology.

[18] Ajani JA, D’Amico TA, Almhanna K, Bentrem DJ, Chao J, Das P, Denlinger CS, Fanta P, Farjah F, Fuchs CS (2016). Gastric cancer, version 3.2016, NCCN clinical practice guidelines in oncology. J Natl Compr Canc Netw.

[19] Pedrazzani C (2015). Should adenocarcinoma of the esophagogastric junction be classified as gastric or Esophageal cancer, or else as a distinct clinical entity?. Ann Surg.

[20] Kurokawa Y, Takiguchi S, Mori M, Doki Y (2015). Surgery for esophagogastric junction tumor. Nihon Shokakibyo Gakkai Zasshi.

[21] Kwon IG, Son YG, Ryu SW (2016). Novel intracorporeal esophagojejunostomy using linear staplers during laparoscopic total gastrectomy: π-shaped esophagojejunostomy, 3-in-1 technique. J Am Coll Surg.

